# 

**Published:** 1886-12-25

**Authors:** 


### THE HOSPITAL

An Institution, Family, and Parochial Journal of
Hospitals, Asylums, & all Agencies for the Care of the Sick, Criticism & News.

### CONTENTS.

**Our First Christmas Day** - - - - 209

**The Story of the Hospitals.** By ONE OF THE PUBLIC.—The ROYAL FREE HOSPITAL - - - 210

**Santa Claus' Garner** - - - - - 212

**Words of Consolation.**—XIII. Sympathy - - 213

**Sister Olive:** A Hospital Romance.—By W. J. ECCOTT 214

**Notes and News.**—Miscellaneous—Medicine Bottles for Hospitals.—The Fleming Memorial Hospital.—The Workington Infirmary.—Dictionaries for Hospitals.—The Surgical Aid Society.—The Burnley Mills and Workshops.—New Hospital for Dublin.—Bazaars.—Hospital Imposture.—Bequests.—Tradesmen's Presents.—Vacancies.—Mercer's Hospital, Dublin.—Penzance Convalescents' Home.—Hospital Collections.—New Cottage Hospital for Ripon.—The Royal Albert Hospital, Devonport.—Bazaar and Fancy Fair at the Rink, Dublin.—Presentation to Mrs. Mercier, etc. etc. - - - - - - - 216

**A Hospital Matron on Hospital Morals** - 218

**The Classes and the Masses: A Word to the Wise.**—II. A Christmas Appeal: Help the Hospitals. By the Ven. ARCHDEACON FARRAR, D.D. - - 219

**Incidents of Animal Life.** Collated by the Rev. F. O. MORRIS, B.A. Stories about Rats - - - 221

**The Editor's Letter-Box.**—National Hospital for Paralysed and Epileptic - - - - - 222

**Disorders of Digestion.**—First Notice.—Introductory - - - - - - - 223

**Isolation Huts:** An Inquiry from America - - 223

**In- and Out-Patients' Hospital Diary** - - 224

**The Children's Corner.**—Puzzles, Answers, etc. - vi

**The Hospital Charity Box.**—Prizes and Results - vi

